# Glucose-stimulated insulin response in pregnant sheep following acute suppression of plasma non-esterified fatty acid concentrations

**DOI:** 10.1186/1477-7827-2-64

**Published:** 2004-09-07

**Authors:** Timothy RH Regnault, Hutton V Oddy, Colin Nancarrow, Nadarajah Sriskandarajah, Rex J Scaramuzzi

**Affiliations:** 1 Commonwealth Scientific and Industrial Research Organization, Division of Animal Production, Prospect, NSW Australia; 2 Beef Cooperative Research Council, Armidale, NSW Australia; 3 School of Environment and Agriculture, University of Western Sydney, Richmond, NSW Australia

## Abstract

**Background:**

Elevated non-esterified fatty acids (NEFA) concentrations in non-pregnant animals have been reported to decrease pancreatic responsiveness. As ovine gestation advances, maternal insulin concentrations fall and NEFA concentrations increase. Experiments were designed to examine if the pregnancy-associated rise in NEFA concentration is associated with a reduced pancreatic sensitivity to glucose in vivo. We investigated the possible relationship of NEFA concentrations in regulating maternal insulin concentrations during ovine pregnancy at three physiological states, non-pregnant, non-lactating (NPNL), 105 and 135 days gestational age (dGA, term 147+/- 3 days).

**Methods:**

The plasma concentrations of insulin, growth hormone (GH) and ovine placental lactogen (oPL) were determined by double antibody radioimmunoassay. Insulin responsiveness to glucose was measured using bolus injection and hyperglycaemic clamp techniques in 15 non-pregnant, non-lactating ewes and in nine pregnant ewes at 105 dGA and near term at 135 dGA. Plasma samples were also collected for hormone determination. In addition to bolus injection glucose and insulin Area Under Curve calculations, the Mean Plasma Glucose Increment, Glucose Infusion Rate and Mean Plasma Insulin Increment and Area Under Curve were determined for the hyperglycaemic clamp procedures. Statistical analysis of data was conducted with Students *t*-tests, repeated measures ANOVA and 2-way ANOVA.

**Results:**

Maternal growth hormone, placental lactogen and NEFA concentrations increased, while basal glucose and insulin concentrations declined with advancing gestation. At 135 dGA following bolus glucose injections, peak insulin concentrations and insulin area under curve (AUC) profiles were significantly reduced in pregnant ewes compared with NPNL control ewes (p < 0.001 and P < 0.001, respectively). In hyperglycaemic clamp studies, while maintaining glucose levels not different from NPNL ewes, pregnant ewes displayed significantly reduced insulin responses and a maintained depressed insulin secretion. In NPNL ewes, 105 and 135 dGA ewes, the Glucose Infusion Rate (GIR) was constant at approximately 5.8 mg glucose/kg/min during the last 40 minutes of the hyperglycaemic clamp and the Mean Plasma Insulin Increment (MPII) was only significantly (p < 0.001) greater in NPNL ewes. Following the clamp, NEFA concentrations were reduced by approximately 60% of pre-clamp levels in all groups, though a blunted and suppressed insulin response was maintained in 105 and 135 dGA ewes.

**Conclusions:**

Results suggest that despite an acute suppression of circulating NEFA concentrations during pregnancy, the associated steroids and hormones of pregnancy and possibly NEFA metabolism, may act to maintain a reduced insulin output, thereby sparing glucose for non-insulin dependent placental uptake and ultimately, fetal requirements.

## Background

To meet the increasing fetal demands and maternal energy requirements of pregnancy, alterations in the partitioning and utilization of maternal nutrients must occur. These adaptations are regulated by changing blood concentrations of regulatory metabolites and hormones, together with changes in target tissue responsiveness. Of principle interest are alterations in maternal glucose metabolism during pregnancy, as glucose is a major limiting nutrient of fetal growth [1,2]. As sheep pregnancy advances, circulating maternal insulin concentrations decline [3,4] and the insulin response to a glucose load is significantly reduced [5,6]. These decreased insulin concentrations during the last third of gestation and into lactation in ruminants have been postulated to be the result of the decreased response of the pancreas to insulinotropic agents [7]. Reducing insulin secretion during pregnancy is proposed to be beneficial to fetal well-being, through the creation of an environment which supports minimizing peripheral glucose utilization and maximizing glucose extraction of the gravid uterus [8,9]. Additionally, the sensitivity of peripheral tissues to insulin is reduced [10,11] and increased mobilization of adipose tissue to supply non-esterified fatty acids (NEFA) as an alternative maternal energy source occurs [12-15]. This pregnancy induced mobilization of adipose tissue is accompanied by a decline in lipid synthesis, and occurs as a result of alterations in insulin receptor numbers, a decreased circulating insulin concentration, as well as interactions with specific hormones of pregnancy [16,17].

High plasma NEFA concentrations have been associated with the development of insulin resistance in the peripheral tissues and also the β-cell [18]. Elevated NEFAs in the muscle inhibit glucose disposal through interactions with the insulin signaling pathways, as certain lipid species act as secondary messengers (ceramide, diacylglycerol and hexosamine), inhibiting insulin signaling [19,20]. Altered serine/threonine phosphorylation of insulin substrate-1 and direct inhibition of components such as protein kinase B, are also sites of action through which NEFAs may give rise to decreased insulin signaling [20,21]. Several of these steps, including the insulin receptor substrate -1 association with phosphatidylinositol, are reduced in liver and muscle during pregnancy [22]. Furthermore, cardiac function is impaired in situations of elevated ceramide, through apoptotic pathways [23]. With regards to the pancreas, elevated NEFA concentrations in rats have been reported to decrease pancreatic responsiveness, resulting in a reduction in glucose stimulated insulin secretion [24-26]. In studies with male rat pancreas, following 48 hours of incubation with elevated NEFA concentrations, glucose-induced insulin secretion, as well as proinsulin biosynthetic responses, are reduced [24,26]. In addition, there is a body of evidence which suggests that chronically elevated NEFA concentrations have a lipotoxic effect on the pancreas, through the formation of nitric oxide and β-cell apoptosis [18,19,27,28].

Studies investigating a possible interaction between increasing NEFA concentrations and maternal pancreatic sensitivity during ovine pregnancy have not yet been reported. The following experiments were designed to examine whether the pregnancy-associated rise in NEFA concentration is associated with a reduced pancreatic sensitivity to glucose *in vivo*. Reported here are *in vivo *pregnant sheep studies of insulin responsiveness to glucose, measured using bolus injection and hyperglycemic clamp techniques.

## Methods

### Animals and experimental design

Twenty-four 3–4 year old, pen-trained Merino ewes of a known gestational age were used. The experiments were conducted adhering to the National Health and Medical Research Council (NH&MRC) guidelines as administered and approved by the University of Western Sydney, Hawkesbury Animal Care and Ethics Committee and the Commonwealth Scientific Research Organization, Division of Animal Production, Animal Care and Experimentation Ethics Committee. All animals displaying estrus following a program of synchronised mating induced by pre-treatment with intravaginal progestogen pessaries were recorded. Pregnancy was later verified by rectal ultrasound scanning at 28 dGA, and then confirmed together with litter size determination at 65 dGA, using abdominal ultrasound. Those ewes displaying estrus, but failing to conceive were used in a non-pregnant, non-lactating (NPNL) control group. A total of 15 NPNL ewes and nine pregnant ewes, three single bearing and six twin bearing, were used in this study. At 90 dGA a trial glucose bolus injection study was conducted in four ewes. At 105 dGA, all animals were studied (total n = 9), in both glucose bolus and hyperglycaemic protocols and then subsequently again at 135 dGA. At each gestational age, NPNL ewes (n = 15) were also studied under both protocols, expect at 90 dGA were only the bolus studies were conducted and only 5 NPNL animals studied.

One week prior to the commencement of the experimental period, ewes were weighed. Body weights (BW) were used to determine the glucose bolus doses to be administered at study (0.4 g glucose/kg). Animals were then individually penned under natural lighting conditions and fed 700 g/d per animal, of a 60:40 pelleted ration, consisting of 60% hammer milled lucerne and 40% hammer milled oaten chaff (92.7 ± 0.34 % dry matter (DM), 68.9 ± 1.05% digestible organic matter, and having a calculated metabolizable energy content of 10.33 ± 0.2 MJ/kg DM, crude protein content of 13.8 ± 0.5%). Water was freely available at all times.

### Glucose Treatments

The ewes were subjected to an experiment protocol consisting of two procedures, 1) a bolus glucose study and 2) a hyperglycaemic clamp study. Each experimental period extended over 5 days, with 2 days in between the procedures. On the day before the commencement of the bolus glucose study, each ewe had polyvinyl jugular vein catheters inserted bilaterally under local anaesthesia. Catheters were maintained patent with daily-heparinized saline flushes (35 U heparin/ml). The bolus glucose procedure was conducted as follows. On the day of the experiment, following a 24-h fast, two pre-injection (basal concentration) blood samples were collected 15 min apart. Immediately following the second basal sample, a glucose bolus was administered. Seven two ml blood samples were collected at 5, 10, 20, 35, 55, 155 and 215 min post-injection and stored in an ice bath until centrifuged at 1,200 × g for 15 min at 4°C. A 0.5 ml aliquot of supernatant was removed for glucose determination, while the remaining plasma was frozen at -20°C for later biochemical analysis.

Two days after the bolus glucose injection study, animals were also subjected to a 120 min hyperglycaemic clamp procedure, which was imposed after a 24 h fast [29]. Prior to the commencement of the clamp, three pre-infusion samples were taken 15 min apart (T -30, T -15 and T 0) through the right jugular catheter. At T 0, a bolus glucose injection was administered through the left jugular catheter and then individual peristaltic pumps were activated to begin the glucose infusion through the left jugular catheter. Blood samples (3.0 ml) were then collected every 5 min from the right jugular catheter. To maintain glucose at the bolus injection level, a spot sample of blood was taken from each 5 min sample and glucose concentration determined using a Boehringer Mannheim Accutrend^® ^blood glucose monitoring kit (Boehringer Germany). The pump speed setting was then adjusted to maintain the required blood glucose concentration. Samples were collected for analysis as described for the bolus protocol.

### Assays and calculations

The plasma concentrations of insulin, growth hormone (GH) and ovine placental lactogen (oPL) were determined by double antibody radioimmunoassay as previously described [30]. Insulin measurements were made for every collection point, while GH and oPL were determined from the pooled pre-bolus samples. Plasma glucose and NEFA concentrations were determined as previously described [31,32]. While glucose samples were determined at each sample point, samples for NEFA concentration during the hyperglycaemic clamp studies were collected at T -30 and T 0 and at 85 and 90 min relative to the imposition of the clamp. Inter-assay and intra-assay co-efficients of variation for low and high quality control samples for all assays are detailed. *Insulin; *Inter-assay LQC, 5.2, HQC, 3.9 and intra-assay LQC, 5.7, HQC, 7.3. *GH; *Inter-assay LQC 10.3, HQC 7.9, and intra-assay LQC 8.6, HQC 7.5.*oPL; *Inter-assay LQC 9.1, HQC 12.0, and intra-assay LQC 9.2, HQC 10.9. *Glucose; *Inter-assay LQC 10.1, HQC 9.3, and intra-assay LQC 10.3, HQC 11.9. *NEFA; *Inter-assay LQC 11.4, HQC 7.8, and intra-assay LQC 10.6, 6.7.

Data from the bolus glucose injection studies were expressed using the following parameters;

- Area under curve (AUC), for both the glucose ((mg/ml)/min) and insulin ((ng/ml)/min) response profiles,

- Glucose clearance rate (CLR; ml/min)

- Glucose half life (t1/2, min)

AUC for both the glucose and the resulting insulin response profiles were calculated using the trapezoidal rule. Glucose clearance rate (CLR, ml/min) was calculated as Glucose dose /AUC. Glucose and insulin half-life was determined from the interpolated cumulative response curve. Glucose and insulin concentrations at 10 minutes were used as the peak response. In the hyperglycaemic clamp studies, in addition to AUC calculations for the whole study period, the Mean Plasma Glucose Increment (MPGI, mg/dl), Glucose Infusion Rate (GIR; g (glucose)/kg BW/min) and Mean Plasma Insulin Increment (MPII; ng/ml) were determined over the last 40 min of the clamp [29], during the GIR steady state period. These results were averaged for each animal, and then pooled to give group results.

### Statistical analyses

There was no significant time of experiment effect when comparing NPNL ewe data collected at each of the gestational age studied, and as a consequence NPNL data from studies at 90, 105 and 135 dGA were pooled and analysed as a single control group against each gestational age group. No differences were found between the singleton and twin bearing ewes and litter size data were pooled to give one pregnant animal group at 105 and 135 dGA (n = 9). Glucose data from the 90, 105 and 135 dGA bolus glucose and 105 and 135 dGA hyperglycaemic clamp experiments were analysed through repeated measures ANOVA with Greenhouse Geisser adjustment [33]. Between ewe variation was included in the ANOVA model. In the hyperglycaemic clamp studies T0 and baseline measurements were used as co-variates. Glucose and insulin AUC, peak concentrations, t1/2 and CLR data from the bolus glucose injection studies were further analysed using the following orthogonal contrasts of a), NPNL versus pregnant ewes; b) day 90 versus day 105 and day 135 ewes and c) day 105 versus day 135. Body weights, glucose doses, insulin, GH, oPL and NEFA concentrations and the derived variables of GIR, MPII and MPGI were all analysed using two sample unpaired Student's *t*-tests. Changes in NEFA concentrations following hyperglycaemic clamp treatment at three different physiological states, NPNL, 105 dGA and 135 dGA was analysed using 2 way ANOVA.

## Results

### Pre experimental body weights, glucose and insulin concentrations

There were no significant differences in body weight (Mean weight: 48.4 ± 1.8 kgs) and required glucose bolus injection (19.5 ± 0.7 g) across the physiological states studied. In the bolus glucose experiments, the basal glucose concentration was significantly lower in pregnant ewes measured on day 135 than on day 90 of gestation (p < 0.05). Basal glucose concentrations in the clamp studies, at 105 and 135 dGA, were also significantly reduced compared to NPNL concentrations (p < 0.05, Table 1). The basal insulin concentrations as gestation advanced declined, and basal levels measured at 105 and 135 days gestation were significantly reduced compared to NPNL concentrations in both experimental treatments (p < 0.05, Table 1).

**Table 1:** Basal glucose (mg/dl) and insulin (ng/ml) concentrations for both bolus glucose injection and hyperglycaemic clamp studies at four gestation age groups. Within columns values with different superscripts are significantly different at p < 0.05 and (n) = number of animals.

	**Glucose**		**Insulin**	
	**Bolus**	**Clamp**	**Bolus**	**Clamp**

**NPNL (15)**	46.9 ± 1.7^a^	51.1 ± 1.9^a^	0.89 ± 0.13^a^	1.09 ± 0.10^a^

**90 dGA (4)**	40.1 ± 4.8^ab^		0.64 ± 0.05^ab^	

**105 dGA (9)**	29.1 ± 4.0^bc^	38.6 ± 3.0^b^	0.49 ± 0.11^bc^	0.53 ± 0.06^b^

**135 dGA (9)**	21.3± 1.9^c^	40.6 ± 4.9^b^	0.29 ± 0.07^c^	0.36 ± 0.06^b^

### Bolus glucose studies

**Figure 1:**
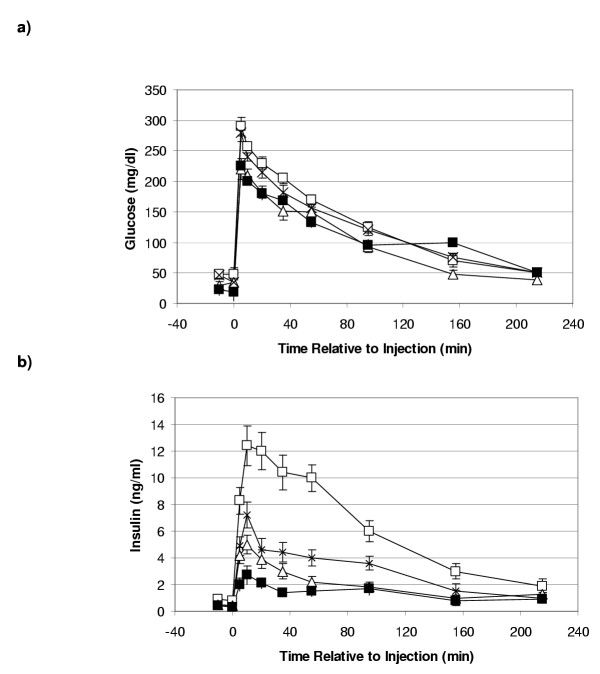
(a) Plasma glucose (mg/dl) and (b) insulin concentrations (ng/ml) following a bolus glucose injection (0.4 g/kg) in NPNL ewes (□, n = 15) and pregnant ewes, 90 (×, n = 4), 105 (Δ, n = 9) 135 (■, n = 9) dGA.

Glucose injection significantly increased glucose concentrations in all groups compared to basal levels (Figure 1a, p < 0.001). After co-variate adjustment for basal glucose concentration, repeated measure ANOVA of glucose concentration after bolus injection revealed no significant differences in maximal glucose concentration obtained between pregnant and NPNL ewes. There was no difference for glucose AUC between NPNL and pregnant ewes, nor did CLR and t1/2 differ between NPNL and pregnant ewes (Table 2).

**Table 2:** Glucose peaks (mg/dl), AUC (mg/ml)/min), clearance rate (CLR, ml/min) and half life (t1/2, min) for NPNL (n = 15) ewes and pregnant ewes, 90 (n = 4), 105 (n = 9), and 135 (n = 9) dGA following bolus glucose injection.

	**Glucose peak**	**AUC**	**CLR**	**t1/2**
**NPNL**	244.9 ± 6.1	166.46 ± 10.2	129.9 ± 7.8	49.6 ± 2.9
**90 dGA**	239.3 ± 21.9	162.0 ± 18.5	124.42 ± 11.9	50.9 ± 3.8
**105 dGA**	192.5 ± 12.1	131.4 ± 9.7	149.1 ± 6.7	48.2 ± 3.2
**135 dGA**	203.4 ± 9.6	162.4 ± 11.4	123.4 ± 8.4	57.4 ± 2.5

Whereas peak insulin concentrations and the insulin AUC profiles were significantly reduced in pregnant compared with NPNL ewes (Figure 1b, p < 0.001 and Table 3). Day 135 ewes had significantly reduced peak insulin responses compared to day 105 ewes (Table 3, p < 0.04). Ewes at 105 and 135 dGA had significantly reduced AUC when compared to day 90 ewes (Table 3, p < 0.001). The decline in AUC was linearly related to stage of pregnancy, whilst insulin t1/2 did not vary significantly across the groups (Table 3).

**Table 3:** Peak insulin responses (ng/ml), AUC ((ng/ml)/min) and half life (t1/2, min) for NPNL (n = 15) ewes and pregnant ewes 90 (n = 4), 105 (n = 9) and 135 (n = 9) dGA following bolus glucose injection.

	**Peak**	**AUC**	**t1/2**
**NPNL**	13.5 ± 1.4	1095.3 ± 112.8	60.7 ± 4.1
**90 dGA**	6.9 ± 2.0	458.8 ± 48.9	61.9 ± 12.2
**105 dGA**	4.7 ± 0.8	277.5 ± 60.3	45.7 ± 7.2
**135 dGA**	2.5 ± 0.5	169.9 ± 24.9	68.9 ± 10.8

### Hyperglycaemic clamp studies

**Figure 2:**
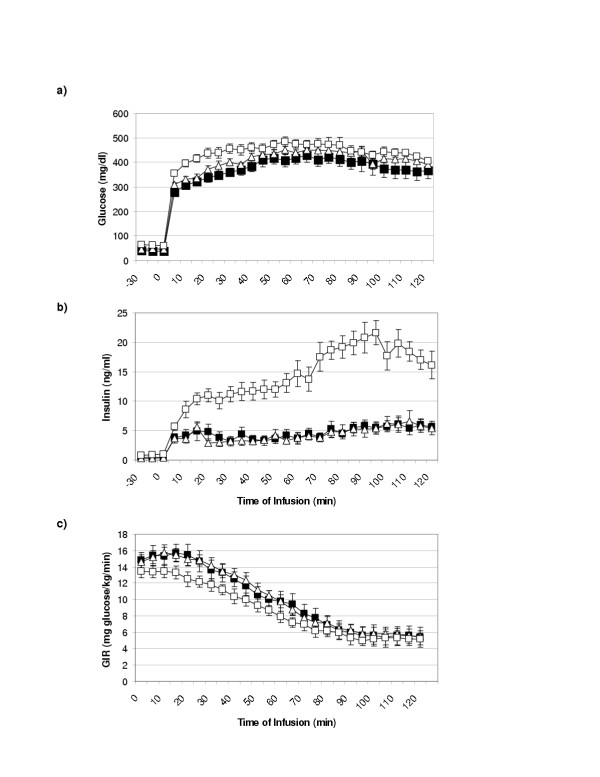
(a) Plasma glucose (mg/dl), (b) insulin concentrations (ng/ml) and (c) glucose infusion rates (mg glucose/kg/min) during a hyperglycaemic clamp conducted on NPNL ewes (□, n = 15) and pregnant ewes at 105 (Δ, n = 9) 135 (■, n = 9) dGA.

The bolus glucose injection, prior to the start of the clamp, significantly elevated glucose concentrations in all groups (Table 1 and Figure 2a, p < 0.001). Following adjustment for basal differences, the glucose concentrations obtained were not statistically different between pregnant and NPNL ewes or between days of gestation. Pregnant ewes while having glucose concentrations not different from NPNL ewes had significantly reduced insulin responses and continued depressed insulin secretion over the 2 hours of the study (Figure 2b, p < 0.001). The measured insulin AUC (ng/ml)/min), were significantly reduced over gestation in 105 dGA and 135 dGA ewes compared to NPNL ewes (NPNL; 1675 ± 110 vs. 105 dGA; 491 ± 67 and 135 dGA; 427 ± 55, p < 0.001).

### Mean plasma glucose increment (MPGI), glucose infusion rate (GIR) and Mean plasma insulin increment (MPII)

The MPGI did not differ significantly between the three physiological states examined (Table 4). Despite a slight rise in glucose concentrations during the clamp, the amount of glucose required to maintain glucose concentrations constant, declined from approximately 14 to 6 mg glucose/kg/min (Figure 2c). A steady state infusion point was obtained in the last 40 min of the clamp. GIR during this period was constant, at approximately 5.8 mg glucose/kg/min, across all physiological states (Table 4). Although MPII during the last 40 min of the glucose hyperglycaemic infusion was significantly greater in NPNL ewes, than in the day 105 and 135 groups (Table 4, p < 0.001), the glucose infusion rates necessary to maintain hyperglycemia within the groups was not significantly altered (Table 4).

**Table 4:** Mean plasma glucose increment (MPGI, mg/dl), glucose infusion rates (GIR, mg glucose/kg/min) and mean plasma insulin increments (MPII, ng/ml) during the last 40 min of hyperglycemic clamp for NPNL (n = 15) ewes and pregnant ewes at 105 (n = 9) and 135 (n = 9) dGA. Within columns values with different superscripts are significantly different at p < 0.05.

	**MPGI**	**GIR**	**MPII**
**NPNL**	368.3 ± 12.5^a^	5.58 ± 0.39^a^	16.44 ± 1.1^a^

**105 dGA**	344.1 ± 24.8^a^	6.03 ± 0.51^a^	5.1 ± 0.61^b^

**135 dGA**	354.4 ± 16.2^a^	5.66 ± 0.71^a^	5.03 ± 0.74^b^

### GH, oPL and NEFA concentrations

**Figure 3:**
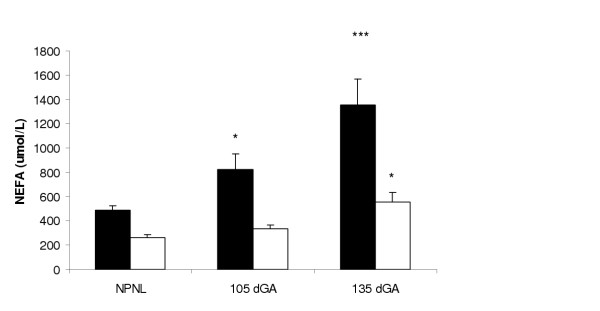
Post fast circulating NEFA concentrations (μmol/L), pre (■) and post (□) the imposition of a hyperglycaemic clamp at three physiological states, NPNL (n = 15), day 105 (n = 9) and day 135 (n = 9) ewes. Comparisons are by 2-way ANOVA compared to NPNL post fasting pre-hyperglycaemic clamp NEFA concentrations, * p < 0.05, **p < 0.01 and *** p < 0.001.

Pre-bolus pooled samples for maternal GH and oPL concentrations displayed increased concentrations with gestational age. Maternal GH concentrations increased with gestation, rising significantly from 0.78 ± 1.1 ng/ml in the NPNL state (p < 0.05), to 2.13 ± 0.35 ng/ml at 105 dGA and 3.5 ± 0.9 ng/ml at 135 dGA. Placental lactogen concentrations were 579 ± 74 ng/ml at 105 dGA and increased to 1211 ± 144 ng/ml (p < 0.05) by 135 dGA. Both pregnant groups displayed higher NEFA concentrations than did the NPNL control ewes following a 24-h fast (Figure 3). Pre-fasting, pre-clamp NEFA concentrations at day 105 were significantly greater than those of NPNL ewes (p < 0.05, Figure 3). Ewes at 135 dGA displayed significantly elevated NEFA concentrations compared to NPNL NEFA concentrations (P < 0.001, Figure 3), and higher concentrations than at 105 dGA (p < 0.06). Following the 2 hour hyperglycaemic clamp, NEFA concentrations were reduced to levels 54% of pre-clamp levels in NPNL ewes, 59% in day 105, and 58% in day 135 ewes (Figure 3). Circulating NEFA concentrations in 105 dGA ewes following the hyperglycaemic clamp were not significantly different from NPNL concentrations, nor were they different from post clamp 135 dGA concentrations, though 135 dGA post clamp concentrations remained elevated above NPNL concentrations (p < 0.05, Figure 3).

## Discussion

There are two important findings of these studies. Firstly, circulating maternal insulin concentration and glucose-stimulated insulin release decrease as gestation advances. Secondly, the imposition of a two-hour hyperglycaemic clamp in pregnant ewes reduces NEFA concentrations, to concentrations not different form the pre clamp concentrations of fasted non-pregnant non-lactating ewes, though despite the reduction, insulin response to glucose remains depressed in 105 and 135 dGA ewes. These results are in agreement with the blunted insulin release during a hyperglycemic clamp treatment observed in lactating cattle, where NEFA concentrations are also elevated late in gestation, prior to a clamp [29,34]. Glucose-stimulated insulin release over gestation in our report was assessed by the capacity of the maternal pancreas to release insulin in response to two forms of glucose challenge. Both of these challenges confirmed that by the end of the second third of gestation (105 dGA, term 147 dGA), a significant and maintained depression in insulin release in the light of elevated glucose levels was observed, which was repeated near term (135 dGA). This suppression of glucose stimulated insulin release was accompanied by increases in maternal NEFA, GH and oPL concentrations.

Lipid metabolism during pregnancy has two distinct phases. The first two thirds of pregnancy are characterised by a low lipolytic activity and the majority of maternal energy appears to be derived from dietary carbohydrate. During the later part of pregnancy and into lactation, there is an increased level of lipolytic activity resulting in increased NEFA concentrations [17], which serve as an alterative energy source, reducing maternal peripheral reliance upon glucose [12,13]. Under these increasing rates of lipolysis, the whole body glycemic response to insulin changes to conserve glucose for uterine uptake and fetal growth [13] and maternal insulin concentrations decline [3,4]. NEFAs are well documented to promote glucose stimulated insulin secretion [35,36,36], however, the chronic effects of elevated NEFA concentrations, specifically as occur during pregnancy, are ill defined, and may be associated with suppressed glucose stimulated insulin release [25]. The responsiveness of the male pancreas has been reported to be decreased following exposure to elevated NEFA concentrations for periods from 3 up to 48 hours [24-26]. In the pregnancy studies presented here, a two-hour hyperglycaemic clamp at two time points in the later half of gestation, reduced NEFA concentrations by approximately 50%, but maternal insulin release in response to glucose remained suppressed. This is in contrast to the same protocol in NPNL ewes, where NEFA concentrations were also reduced, though pancreatic insulin response was maintained. It is interesting to note that the NEFA concentrations before the clamp in NPNL ewes were similar to pregnant ewes' NEFA concentrations at the end of the clamp procedure, and yet the NPNL insulin response remained unaltered and NPNL NEFA concentrations had declined by approximately 50%. In contrast, in the pregnant ewes, insulin release remained suppressed, despite a comparable depression in NEFA concentrations as generated in the NPNL ewes, similar to NPNL pre-clamp NEFA concentrations. Somewhat similar studies have been conducted in fed and fasted non-pregnant mice [36]. When a hyperglycaemic clamp was imposed, NEFA concentrations were reduced. While fasted animals had a normal first phase insulin release, similar to the fed animals, this response fell away and significantly reduced insulin secretion rates were observed [36]. The interesting differences between these mice studies and the pregnant sheep response, is that the initial insulin response is also blunted in pregnancy, suggesting other effects of fasting and or pregnancy are involved in this absolute suppression of a glucose stimulated insulin response.

These data demonstrate that the acute suppression of NEFA concentrations per se during pregnancy does not result in the restoration of a NPNL-like insulin response. This suggests that possibly other factors of pregnancy, such as the influence of lactogenic and steroid hormones, and NEFA metabolism may be acting upon the maternal pancreas to suppress the maternal insulin response during pregnancy in sheep. Placental lactogens have been documented in many mammalian species, including the sheep and human, and PL concentrations increase with advancing gestational age, reaching maximal concentrations just prior to term [37,38], similar to what is reported in this study. Homologous studies with human placental lactogen, prolactin and growth hormone have shown significantly elevated *in vitro *secretion of insulin from pancreatic islets [39]. Interestingly, more recently it has been suggested that PL and placental GH act in concert to modulate maternal metabolism, resulting in an increase in the available supply of glucose and amino acids to the fetus [9]. Studies concerning the effect sheep PL may have on pancreatic function however, are not definitive [40-42]. The short-term removal of oPL by immunoneutralisation increased insulin concentration [42], while an acute oPL infusion failed to demonstrate any significant changes in insulin levels [41]. However, in the carunculectomy model of fetal growth restriction (FGR), carunculectomised ewes have increased glucose concentration, without any increase in insulin [40], suggesting that there may be a pregnancy-specific impairment of insulin secretion during sheep pregnancy.

Placental progesterone has also been reported to play a major role in modulating insulin release during pregnancy. In rat islet studies, progesterone counteracts lactogenic insulin stimulatory behaviour [43], and circulating progesterone levels in the sheep increase from approximately 50 dGA [38,44]. Possible differential and interactive effects of ovine PL and progesterone on insulin secretion as gestation advances, as observed in the rat [43,45], remain to be fully explored. Despite this fact, changes in adipose tissue insulin sensitivity suggest that in pregnancy, insulin resistance may develop together with a change in the pattern of substrate utilization, which occur under rising progesterone, prolactin, PL and GH concentrations [9,13,46]. Actions of GH on adipose tissue include inhibition of insulin-induced fatty acid synthesis [47]. Also a down-regulation of GLUT-1 and GLUT-4 *in vivo*, in rat adipocyte plasma membranes incubated with GH, has resulted in a reduced adipocyte glucose uptake [48], associated with a down regulation of insulin sensitivity or responsiveness. In addition, despite initial experiments reporting that GH and oPL do not modulate lipolytic activity [17], there is evidence to suggest PL and prolactin decrease adipocyte glucose transport [49] and that oPL may act in concert with GH, enhancing GH effects on the pattern of substrate utilization [9,50].

Another candidate that presents itself as possibly regulating maternal insulin response is that of leptin. Leptin concentrations rise during pregnancy [51], and stimulates lipolysis in muscle tissues [52], and may also be involved in a unique form of lipolysis where glycerol is released instead of NEFAs [53]. A direct role of action upon human and rat islet cells has been demonstrated in culture and *in vivo, *through actions in the regulation of Ca^2+ ^influx into the β-cell [54,55]. However, when leptin was administered to fasted mice there were no observed changes in plasma insulin or glucose concentrations [54], suggesting other factors in a fasted state act to regulate pancreatic insulin release in response to glucose. Leptin concentrations were not determined in the study reported here, but the relationship between elevated leptin levels during pregnancy and pancreatic responsiveness is an interesting one, and one that requires further study *in vivo*. One final possible explanation of the data reported here could be that the chronic elevation in NEFA levels, as occurs in pregnant ewes, does play a role. While the acute suppression of NEFA concentrations during pregnancy did not result in the restoration of a NPNL-like insulin response, a chronic exposure to elevated NEFAs imparts a continuous 'lipotoxic' inhibitory effect. Increased NEFA concentrations have been associated with a lipotoxic effect on the pancreas resulting in suppressed insulin release and β-cell apoptosis, following the formation of nitric oxide [18,19,27,28], possibly similar to that demonstrated to occur in myocardium [23]. However no studies have yet been conducted to examine this possible pathophysiological outcome in the pregnant animal.

One final aspect of the present studies was the attempt to use the hyperglycemic clamp parameters of MPGI, GIR and MPII [29,34], to define more precisely pregnancy-induced insulin resistance in sheep. The MPGI was constant across all physiological states and MPII, as an index of the ewe's pancreatic responsiveness to elevated glucose concentrations, was reduced in late pregnancy, reflecting the depressed sensitivity of the pancreas throughout the hyperglycemic clamp. During the final 40 minutes of the hyperglycemic clamp, GIR achieved glucose concentrations remained similar between the groups, suggesting both NPNL ewes and pregnant ewes were utilizing and extracting available glucose at the same rate, though to obviously different sinks. This is further supported by the fact that following a fast, both NPNL and pregnant ewes had glucose clearance rates not significantly different from one another. In the present experiments, feed intakes were not different across pregnant and non-pregnant ewes and the constant infusion rates suggest similar total utilization rates, while the difference in MPII reflects different patterns of glucose utilization. Thus as pregnancy advances, the site of utilization changes, from peripheral tissues (muscle and adipose tissues) to the gravid uterus [13,56]. If there were no mechanisms in place to suppress peripheral glucose utilization, be it insulin resistance or reduced insulin output, it should be expected that pregnant animals would show a greater GIR and an increased MPII. Therefore, as GIR was constant across groups and insulin secretion is suppressed, the peripheral glucose utilization, through the induction of peripheral insulin resistance, must be depressed in an effort to support glucose homeorhesis during pregnancy.

## Conclusions

Increasing NEFA concentrations as gestation advances provide the maternal compartment with a double advantage. Firstly they act to an alterative fuel source for maternal metabolism. Secondly, they act to promote the development of an insulin resistant state, which is aided through the suppression of the glucose-stimulated insulin release response. This later action is still not well defined in pregnancy, but could be the result of interactions between hormones of pregnancy and the maternal pancreas, and possibly the result of chronically elevated NEFA concentrations. Through the development of insulin resistance in maternal peripheral tissues and a reduced insulin output, glucose is 'spared' and available for placental uptake and ultimately, fetal demands.

## List of abbreviations as defined in the text

NEFA Non-esterified fatty acids

NPNL Non-pregnant, non lactating

BW Body weight

DM Dry matter

GH Growth hormone

OPL Ovine placental lactogen

AUC Area under curve

CLR Glucose clearance rate

MPGI Mean plasma glucose increment

GIR Glucose infusion rate

MPII Mean plasma insulin increment
